# The Relationship between a Defined Microstructure within the Mold Surface and the Corresponding Roughness on the Part: A Systematic Study on Particle Size, Filler-, and Compatibilizer Content

**DOI:** 10.3390/polym13162757

**Published:** 2021-08-17

**Authors:** Roman Christopher Kerschbaumer, Silvester Bolka, Teja Pesl, Ivica Duretek, Thomas Lucyshyn

**Affiliations:** 1 Polymer Competence Center Leoben GmbH, Roseggerstrasse 12, 8700 Leoben, Austria; 2 Faculty of Polymer Technology, Ozare 19, 2380 Slovenj Gradec, Slovenia; silvester.bolka@ftpo.eu (S.B.); teja.pesl@ftpo.eu (T.P.); 3 Montanuniversitaet Leoben, Department of Polymer Engineering and Science, Polymer Processing, Otto Gloeckel-Strasse 2, 8700 Leoben, Austria; ivica.duretek@unileoben.ac.at (I.D.); thomas.lucyshyn@unileoben.ac.at (T.L.)

**Keywords:** haptic properties, surface topography, molded surface roughness, mold insert roughness, impression quality, replication, filler content, mean filler size, compatibilizer content, microstructure

## Abstract

The perception of a surface and its haptic properties are significantly influenced by roughness and microstructure, respectively, whereby non-negligible parameters include friction, contact area, temperature, and humidity between the human finger and the examined surface. In particular, for a scientific investigation on haptic influences, the production of samples with a defined surface roughness is indispensable. The aim of this study is to analyze the impact of various mold insert roughnesses combined with the influences of particle size, filler-, and compatibilizer content on impression quality. An unfilled high density polyethylene was chosen as a reference for the impression quality investigations, while fillers with significantly different particle sizes and a compatibilizer were used to produce proprietary compounds. Injection molded parts were manufactured utilizing mold inserts with three different line roughness values. To support the obtained results, a multivariate analysis of variance, a simulation of the filling phase as well as a rheological material characterization were conducted. The results revealed that (i) the impression quality can be independent of the applied insert roughness based on the filler particle size that was studied, (ii) an increasing on both filler particle size and compatibilizer content raise the sample roughness as a function of the penetration ability of the filler into the insert valleys, and (iii) with a higher insert roughness, the thermoplastic moldings generally exhibit a significantly smoother topography. An assumed correlation between part roughness and melt viscosity could not be confirmed.

## 1. Introduction

Besides the product design and its application-related characteristics, haptic properties play a decisive role, especially in the case of visible products, i.e., products that are touched by human beings. Spence et al. formulated the importance of touch-feel sensation in a broad economic and social sense. Based on former knowledge and cognitive perception, a particular product might not be considered if the previously suggested expectation does not correlate with the real haptic experience [1]. In general, the topography of a polymer part surface will be customized during production or via post-treatments. Additional application steps, e.g., a plasma treatment before coating [2,3] and metallization [4] will be avoided if possible, as they increase the product price. Wettability is a key parameter for detecting changes in surface properties, i.e., the resulting angle between a test liquid and a surface under investigation. For this purpose, contact angle measurements are usually performed either with the sessile-drop or with the modified Wilhelmy balance technique [5,6,7]. But in contrast to ideal surfaces [8], the wettability of a product surface is significantly influenced by the component roughness [9]. However, injection molding is a common manufacturing method for parts with large dimensions exhibiting surface microstructures in a more economical manner, i.e., in large quantities at manageable costs [10]. Numerous research groups are working on impression quality or replication rate to identify influences of process parameters [10,11,12,13,14,15], wear or damage of mold steel [16], and material behavior of the applied thermoplastics [17,18]. The following literature survey reveals that the most important parameters influencing impression quality differ considerably. Both, melt and mold temperature are indicated as the main influences on impression quality. A higher melt temperature of the processed thermoplastic causes a decrease in the material viscosity, resulting in an improved flow behavior [17]. Thus, materials exhibiting low levels of viscosity at given process conditions are preferred when micron-scale structures will be molded, in order to suppress a premature solidification of the flow front. In this context, Rytka et al. [18] observed in their study that the solidification time of the flow front during the filling process of v-shaped microgrooves with an average structure width of 22 μm and a maximum height of 123 μm ± 6 μm, corresponding to an aspect ratio of 5.6, is in the range of milliseconds. Moreover, the no-flow temperature for smaller structures will be reached sooner, due to the comparatively small volume required during texture impregnation. Accordingly, energy will be transferred rapidly from the volume element in contact with the wall into the cold mold by heat conduction. This effect can be delayed by raising the mold temperature. Nevertheless, with a standard temperature control of the mold, the process times will be increased, since higher mold temperatures require longer cooling times. To compensate it, a rapid heat cycle molding process, as described by Pacher [15], can be considered. Wang et al. [11] proposed a critical mold surface temperature prior to contact with filled polymer systems to inhibit filler exposure. Typically, only minor influences are exerted on the impression quality by injection rate as well as holding pressure [10,13,17]. Besides the process parameters, the topography will be influenced by additives such as fillers or compatibilizers. Polymer melts containing fillers tend to cool down faster, since the addition of fillers generally increases the thermal conductivity [19,20]. As a result, both, the heat transfer coefficient and the viscosity level change [10,12,18,21]. Kuroda et al. [22] investigated the influence of talcum filler size on surface roughness and assumed an effect on the replication of microstructures. Subsequently, compatibilizers have been used to improve the bonds between the polymer matrix and the filler, leading to an increase in mechanical properties of the final product. During the compounding process, however, the compatibilizer is grafted to the filler, which generally increases the filler dimension. Therefore, a grafting reaction might affect the impression quality in a similar way as does an increase of the filler particle size [23].

To summarize, the perception of a surface and its haptic properties are significantly influenced by roughness and microstructure, respectively, whereby non-negligible parameters include friction, contact area, temperature, and humidity between the human finger and the examined surface [24]. In particular, for a scientific investigation on haptic influences, the production of samples with a defined surface structure is indispensable. This study aims to analyze the impact of various mold insert roughnesses combined with the influences of filler particle size, filler-, and compatibilizer content on impression quality. To support the obtained results, a multivariate analysis of variance, a simulation of the filling phase as well as a rheological material characterization will be conducted.

## 2. Materials and Methods

### 2.1. Materials

Preliminary investigations were carried out using the following commercially available thermoplastic materials: (i) a high density polyethylene (HDPE) copolymer SABIC^®^ HDPE CC2056 applied as a reference as well as for the in-house production of different compounds and two compounds based on polyamide (PA6) resins showing high thermal conductivity. The PA6 based resins include (ii) LNP™Konduit™PX11313 containing mineral filler as well as glass fiber and (iii) LNP™Konduit™PX13012 containing only mineral filler. All three materials were purchased from SABIC AG, Riad, Saudi Arabia. Finally, (iv) a thermally conductive, low density, and heat resistant polycarbonate (PC) grade Makrolon^®^ TC8030 from Covestro AG, Leverkusen, Germany, as well as (v) a highly filled polyamide (PA) resin UNITAKA Thermal Conductive Resins N2003-M NT-NM2, UNITAKA Ltd., Osaka, Japan, was used. For the in-house produced compounds, three fillers with significantly different top cut and median particle size values, respectively, were applied to investigate the influence of filler content as well as particle size on the roughness of the molded part. These fillers are (i) CalPlex Extra (Calcit d.o.o., Stahovica, Slovenia), (ii) Plustalc H15 (Caldic Vietnam Co. Ltd., Ho Chi Minh, Vietnam), and (iii) CalPlex 40 (Calcit d.o.o., Stahovica, Slovenia). The physical properties of the considered filler regarding the particle size distribution are shown in Table 1. However, the geometry of the fillers, e.g., spherical, elliptical, or platelet-shaped are not specified by the supplier, and will not be further investigated in this study. To increase the homogeneity of the in-house produced compounds, e.g., HDPE with different content of filler, a maleic anhydride functionalized high density polyethylene (PE-g-MA) compatibilizer Exxelor™PE 1040 (ExxonMobil Petroleum and Chemical, Machelen, Belgium) was introduced.

### 2.2. Compounding of Different Formulations

Six HDPE compounds per filler type were produced on a twin screw extruder LTE20–44 (LABTECH Engineering Company Ltd., Samut Prakan, Thailand) with a screw diameter of 20 mm, a length-to-diameter (L/D)-ratio of 44:1, and a subsequent pelletizing unit. The corresponding settings are listed in Table 2 and Table 3. Moreover, aiming to obtain samples with the same processing history, the neat HDPE was processed once with the help of the twin screw extruder.

### 2.3. Manufacturing of Injection Molded Parts

**Figure 1:**
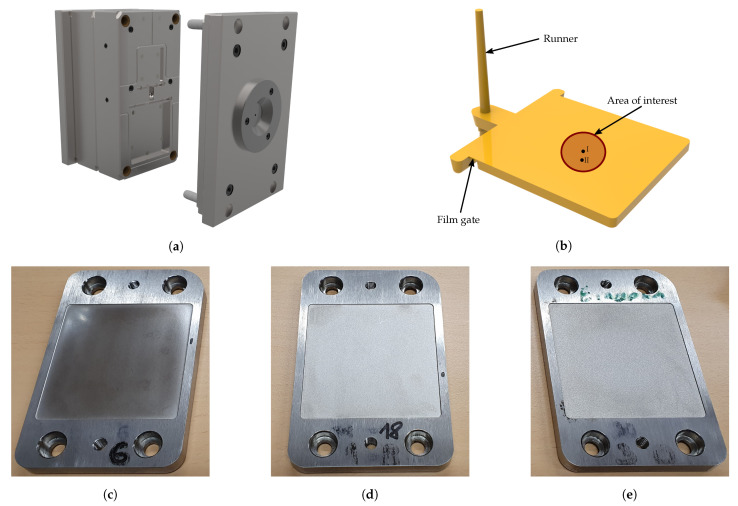
Applied equipment for the production of injection molded parts with specified roughness values. (**a**) Mold with interchangeable inserts. (**b**) Injection molded part with a dimension of 60 mm × 60 mm × 4 mm applying an insert without a specified roughness. (**c**–**e**) Provided mold inserts from Richard Hiebler GmbH., Stainz, Austria, with target line roughness values $R_{a}$ of 0.2 μm, 0.8 μm, and 3.2 μm to produce rectangular shaped parts with a dimension of 60 mm × 60 mm × 2.5 mm.

Prior to the production of injection molded parts, the granulate needs to be pre-dried, since the compounding step including subsequent pelletizing results in an increased moisture content. In general, excessively moist material generally leads to an impaired spectrum of overall properties in the molded part, i.e., not only the mechanical but also the optical, chemical, and physical properties are affected [25]. Obvious defects will be streaks, cavities, holes as well as bubbles on the surface. To prevent these defects, the granulate was dried in an IFE 500 dryer (Memmert GmbH. + Co. KG, Schwabach, Germany) at a temperature of 80 °C for 4 h. The level of relative humidity reached for all formulations was checked with a moisture analyzer HX 204 (Mettler Toledo GmbH., Greifensee, Switzerland) after drying, whereby the relative humidity level was below 0.1%. Afterwards, the specimens required for the investigation of the roughness were produced with commercially available thermoplastics as well as with in-house produced compounds on a CX50-180 injection molding machine (KraussMaffei Group GmbH., Munich, Germany) with a screw diameter of 30 mm and a mold with exchangeable inserts. One standard insert, i.e., without a specified roughness, and three other exhibiting a target line roughness of 0.2 μm, 0.8 μm, and 3.2 μm are implemented (Figure 1). All samples with HDPE-matrix were injection molded with the same settings. For the filled and commercial thermoplastic materials, the manufacturers’ recommendations were selected for the preparation of the injection molded samples, as shown in Table 4.

### 2.4. Termogravimetric Analysis

Thermogravimetric analysis (TGA) was performed on a TGA/DSC 3+ STAR^e^ thermal analysis (Mettler-Toledo GmbH., Greifensee, Switzerland) to estimate the filler content of the commercially available thermoplastics, since these values were not provided. The analyses were performed in a nitrogen atmosphere with a purge rate of 20 mL min${}^{-1}$ in the temperature range of 40 °C to 550 °C, with a heating rate of 10 K min${}^{-1}$. After reaching the maximum temperature, all samples were kept in an oxygen atmosphere, i.e., purge rate of 20 mL min${}^{-1}$, for 30 min to obtain the inorganic components (filler content) as residue [26]. Moreover, only one analysis per sample was carried out to estimate the filler content.

### 2.5. Deployed Material Characterization Methods Required for the Simulation of the Filling Phase

To determine the material properties required for a filling simulation, the methods described below were deployed.

The viscosity (η) of the formulations was analyzed with a modular compact rheometer MCR 702 MultiDrive (Anton Paar GmbH., Graz, Austria) in oscillating mode with plate-plate geometry of 25 mm diameter in accordance with ISO 6721 [27]. Frequency sweep measurements in the range from 0.1 rad s${}^{-1}$ to 500 rad s${}^{-1}$ were performed at strains in the linear viscoelastic range at temperatures of 175 °C, 195 °C, and 215 °C. Since the Cross-Williams-Landel-Ferry (Cross-WLF) model is the most common viscosity model employed by injection molding simulation software, it was chosen, because it offers the best fit to most viscosity data [28,29]. The approximation as well as temperature shift of viscosity are given in Appendix A.

Specific heat capacity ($c_{p}$) measurements were carried out according to ISO 11357 [30] with a differential scanning calorimeter DSC 1 STAR^e^ System (Mettler-Toledo GmbH., Greifensee, Switzerland) by a heating rate of 10 K min${}^{-1}$ and a cooling rate of 20 K min${}^{-1}$. The sample mass was about 8 mg. To inhibit oxidation reactions during the $c_{p}$-measurement, the experiments were conducted in a nitrogen atmosphere with a purge rate of 50 mL min${}^{-1}$.

In accordance with ISO 17744 [31], the pressure-specific volume-temperature (pvT)-behavior was determined in isobaric cooling mode (0.1 K s${}^{-1}$) between 200 °C and 40 °C at pressure levels between 20 MPa and 140 MPa in 30 MPa steps. The tests were carried out on a PVT-100 device (SWO Polymertechnik GmbH., Krefeld, Germany). Measured pvT-data were approximated with the two-domain pvT-model of Tait [32,33] (see Appendix A).

In the melt state, the thermal conductivity (λ) was characterized with a K-System II device (Advanced CAE Technology Inc., Ithaca, NY, USA) at two temperatures (180 °C, 200 °C) according to ASTM D5930-17 [34] and in the solid state with a TCi (C-Therm Thechnologies Ltd., New Brunswick, Canada) at two temperatures (27 °C, 60 °C) on previously compression molded test plates in accordance with ISO 22007-2 [35] and ASTM D7984-16 [36], respectively. The required compression molded test plates with a thickness of 5 mm and a diameter of 25 mm were prepared on a vacuum press P 200 PV (Dr. Collin GmbH., Maitenbeth, Germany). During the heating phase, a temperature of 195 °C for each of the four phases and a total time of 13 min were selected, while the temperature during the cooling phase was 30 °C for 10 min. For each phase, the applied pressure was raised by 0.1 MPa.

### 2.6. Supported Simulation of the Filling Phase

Supporting simulations of the filling phase were carried out by means of a state-of-the-art injection molding simulation package Moldflow^®^ Insight, release 2019.05 (Autodesk Inc., San Rafael, CA, USA) in order to estimate the occuring shear rates when the polymer reaches the cavity wall. In addition, extensive shear dissipation and thus a significant increase in the melt front mean temperature can be expected, due to the low height of the film gate of about 0.5 mm. Both values are considered important because the shaping, i.e., in particular the impression of the mold surface on the thermoplastic part, takes place during this step. Due to the large number of formulations (19), a simulation of the filling phase as well as a full material characterization was only performed for the unfilled HDPE. To simulate the filling process, the following setup was chosen: (i) the part as well as the mold were meshed with a 3D tetra mesh, selecting 20 elements along the thickness direction of the part, (ii) beam elements were considered for the cooling system, and (iii) by means of a transient cooling simulation, i.e., Cool(FEM) option, the mold temperature was calculated. Based on the simulated shear rates at the mold surface and melt front mean temperature for the unfilled HDPE, comparative viscosity measurements in oscillating mode were done for all other formulations. The aim of this approach is to prove whether the impression quality correlates with the material viscosity.

### 2.7. Determination of the Roughness

Although the surface roughness of an injection molded part is decisive for haptic perception, in technical drawings, e.g., for the manufactured inserts, roughness is typically given as line roughness. Consequently, both, the line roughness ($R_{a}$) and the surface roughness ($S_{a}$) were determined with a confocal microscope Microprof MPR1080 (Fries Research and Technology (FRT) GmbH., Bergisch Gladbach, Germany) and compared for selected parts. The main advantage of the FRT confocal microscope is that transparent or semi-transparent samples can be measured with little effort. Furthermore, the influence of the operator is negligible, since only the measurement length or the measurement range has to be entered. In addition, the recorded signal must be in a reliable range of the detecting sensor. If this can not be ensured, the intensity has to be adjusted. However, the disadvantage is that the surface scanning for determining the surface roughness by the existing FRT device requires a significant amount of time, so that a measurement area of 4 mm${}^{2}$ takes about 12 h. Before an unknown roughness of a specimen can be determined with the FRT, preliminary measurements according to ISO 4288 [37] have to be conducted to distinguish between roughness and waviness. The distinction is based on the cut-off wavelength ($\lambda_{c}$), which is assigned to the waviness (long wavelength), and those to the roughness (short wavelength). Table 5 lists the cut-off wavelength to be selected for non-periodic profiles depending on the expected roughness. With the $\lambda_{c}$ selected, the required measuring length *l* can be calculated as follows:

(1) $$l=7\lambda_{c}.$$

After the preliminary investigations, the roughness was determined at two positions in the middle area of the sample, i.e., on the surface where the film gate is located (cf. Figure 1). For each position, five line measurements with a spacing of 1 mm, and for the surface roughness measuring areas of 0.25 mm${}^{2}$, and 4 mm${}^{2}$, respectively, for selected samples were defined. Due to the limited measurement possibilities at the FRT, e.g., maximum measurement area of 2 mm × 2 mm, cut-off wavelengths deviating from the standard were selected for the area measurements. This is permissible, if the samples to be examined exhibit minor or no waviness. Finally, $R_{a}$ [38] and $S_{a}$ [39] can be calculated by:

(2) $$R_{a}=\frac{1}{l}\int_{x=0}^{l}\left|z(x)\right|\;dx,$$

(3) $$S_{a}=\underset{A}{\;\int \int \;}\left|z(x,y)\right|\;dx\;dy,$$

where *z* is the measured profile along the vector *x* and *A* is the area mapped by the vector *x* and *y*.

To describe other effects, two additional profile parameters will be taken into account. These include $R_{z}$, the average peak-to-valley height of a profile, and *S*, the mean distance between the local (positive) profile peaks. $R_{z}$ [38] and *S* [40] can be written as:

(4) $$R_{z}=\frac{1}{j}\sum_{i=1}^{j}R_{z_{i}},$$

(5) $$S=\frac{1}{j}\sum_{i=1}^{j}S_{i}.$$

In Equations (4) and (5), *j* is the number of peaks, $R_{z_{i}}$ indicates the maximum peak-to-valley height within the interval $\lambda_{c}$, and $S_{i}$ specifies the local distance between two positive peaks.

For evaluating the measurement capability of the applied confocal microscope, reference measurements were conducted on a VDI 3400 reference gauge (Charmilles Technologies S.A., Geneva, Switzerland). This reference gauge consists of 16 samples of spark-eroded surfaces whose roughness values correspond to the numbers 0–45 as specified in the internationally applied guideline VDI 3400 [41]. A label on the reference gauge back indicates the corresponding line roughness value for each surface.

## 3. Results and Discussion

**Figure 2:**
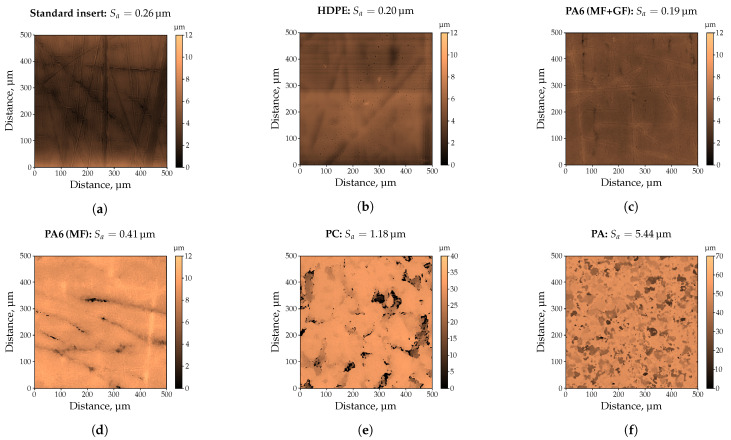
Preliminary investigations on the impression quality of commercially available thermoplastics by means of a confocal microscope. A cut-off wavelength of 71 μm was selected. (**a**) Standard insert: $S_{a}$ = 0.26 μm. (**b**) HDPE: $S_{a}$ = 0.20 μm. (**c**) PA6 containing mineral filler (MF), and glass fiber (GF): $S_{a}$ = 0.19 μm. (**d**) PA6 containing mineral filler (MF): $S_{a}$ = 0.41 μm. (**e**) PC: $S_{a}$ = 1.18 μm. (**f**) PA: $S_{a}$ = 5.44 μm.

For a scientific investigation about the influences on haptics, it is crucial to be able to produce specimens with defined surface properties, such as specified roughness values. Consequently, the injection mold equipped with a standard insert, i.e., without a defined surface roughness, was selected to produce specimens with five commercially available thermoplastic materials in order to investigate a possible influence of the material to be processed on the impression quality. Subsequently, the impression quality of these moldings was examined with a confocal microscope and compared in Figure 2.

**Figure 3:**
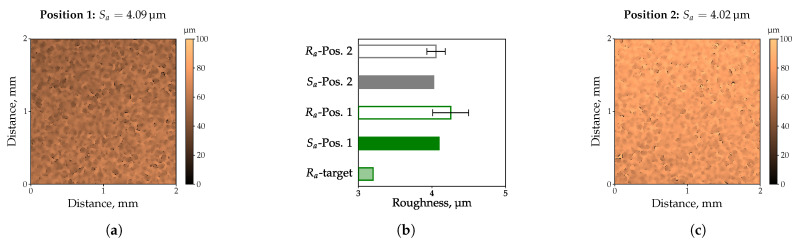
Reproducibility of surface $S_{a}$ and line roughness $R_{a}$ based on insert with the highest target line roughness of 3.2 μm. Topographies of Position 1 (**a**) and Position 2 (**c**) are similar. The determined roughness values are equal but are approx. 30% higher compared to the target line roughness value of the insert (**b**).

**Figure 4:**
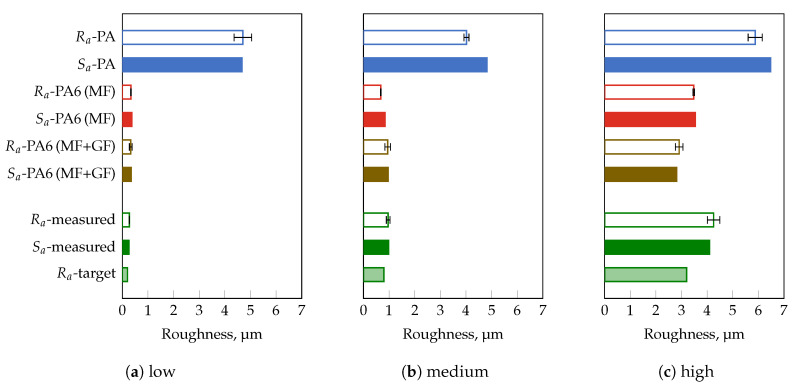
Measured surface $S_{a}$ and line roughness $R_{a}$ values are equal to each other as long as no sample curvature is present. In addition, the impression quality is significantly influenced by the thermoplastic material. Measurement range of 2 mm × 2 mm for $S_{a}$ and according to ISO 4288 [37] for $R_{a}$. Mineral filler (MF) and glass fiber (GF). Deployed insert with a target line roughness of (**a**) 0.2 μm (low), (**b**) 0.8 μm (medium), and (**c**) 3.2 μm (high).

The measured surface roughness of the standard insert deployed is $S_{a}$ = 0.26 μm, whereby scratches in the range of 1 μm–2 μm could be detected (Figure 2a). When using HDPE (Figure 2b) or PA6 (MF + GF), i.e., filled with mineral fillers and glass fibers (Figure 2c), the surface structure of the standard insert could not be completely molded, resulting in lower surface roughness values of $S_{a}$ = 0.20 μm, and $S_{a}$ = 0.19 μm, respectively. As shown in Figure 2d–f, the obtained surface roughness values for the three filled and commercial thermoplastics are significantly higher, i.e., up to a factor of 27 for the highly filled PA compared to the unfilled HDPE. Based on the filler content determined by means of TGA (Table 6), it is obvious that the filler content cannot be the only factor influencing the impression quality. Confirming this, the lower filler content of PC compared to PA6 (MF + GF) would lead to lower surface roughness values, which could not be observed. Similarly, PA6 (MF + GF) and PA6 (MF) exhibit almost the same filler content, whilst a factor of two is detectable in the surface roughness. Instead, it can be assumed that the shape, e.g., almost spherical or with a length-to-diameter ratio, quantity, and size of the used fillers are decisive for how the surface topography of the cavity will be molded. If this assumption meets the results, the use of inserts with different roughness values would lead to even more significant differences in impression quality. For this purpose, the inserts provided by Richard Hiebler GmbH., (Stainz, Austria) with target line roughness values of 0.2 μm (low), 0.8 μm (medium), and 3.2 μm (high) were applied. Before manufacturing the injection molded parts, the actual roughness of the inserts was determined by means of a confocal microscope. As shown in Figure 3, as an example for the insert with a target line roughness of 3.2 μm, the surface roughness agrees very well with the results of the line roughness. Nevertheless, it can be seen that the measured roughness values $S_{a}$ and $R_{a}$ are approximately 30% higher compared to the target line roughness value of the insert. This could also be observed for the other two inserts as proved in Figure 4 (cf. Table 7).

In order to verify the suitability of the confocal microscope as well as to clarify the significant differences between the target and measured roughness values of the selected inserts, measurements were carried out on a VDI 3400 reference gauge with defined $R_{a}$-values, and compared in Table 8. The standard deviation in the investigated measuring range (0.1 μm–3.2 μm) is less than 12%. For the equivalent roughness values of the VDI-gauge and the three inserts (0.2 μm, 0.8 μm, and 3.2 μm), the standard deviation is less than 8%. Compared to the roughness values specified in VDI 3400 reference gauge, the deviation from the measurement results is less than 16%, and lower than 12% for the three roughness values of the inserts. The results prove that the applied confocal microscope is suitable for the determination of the roughness. Furthermore, the difference between the target and measured roughness of about 30% does not originate from the applied confocal microscope. This could be explained by the fact that it is common practice in industry, after producing the desired roughness values, e.g., by means of spark erosion, to check the roughness only with a VDI 3400 reference gauge, i.e., yielding to an accuracy within two VDI classes, or with portable hand-held devices. Finally, the mold was utilized with three inserts, showing significant differences in roughness values to produced parts from three thermoplastic materials with almost the same filler content, e.g., PA6 (MF + GF), PA6 (MF), and PA. Figure 4 compares the obtained surface and line roughness values.

**Figure 5:**
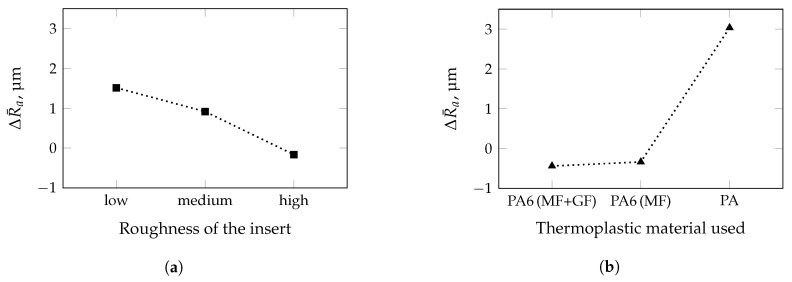
Results of multivariate analysis of variance performed with a confidence interval of 95%. Mineral filler (MF) and glass fiber (GF). Significant change in impression quality $\Delta {\bar{R}}_{a}$ as a function of (**a**) the roughness of the insert and (**b**) the thermoplastic material.

In general, it can be observed that both, $S_{a}$ and $R_{a}$ match if the specimens do not exhibit any curvature. In the case of curvatures due to shrinkage and warpage, it is mandatory to proceed according to ISO 4288 [37] with cut-off wavelengths shown in Table 5. Thus, only the results of the line roughness values will be discussed in more detail. A perfect impression can only be achieved by utilizing the mold with the insert of medium roughness and PA6 (MF + GF) as material. All other combinations of insert roughness and thermoplastic material affect the impression quality, i.e., difference between part and insert roughness. For the two differently filled PA6 grades, the molded roughness is about 20% higher for the insert with the lowest roughness. In contrast to that, an opposite trend for the insert with the highest roughness can be observed. The filled PA reveals almost an independence between the insert and the molded line roughness of the specimen, resulting in the highest $R_{a}$-values of 4.70 μm, 4.02 μm, and 5.88 μm. Hence, a multivariate analysis of variance (MANOVA) was performed to test the influence of the insert roughness and the material on the impression quality. Within a 95%–confidence interval, the insert roughness as well as the thermoplastic material is statistically significant (Figure 5). As illustrated in Figure 5a, applying the insert with the lowest roughness results in average to a higher roughness on the molded part ($\Delta {\bar{R}}_{a}$ = +1.5 μm). Continuing to increase the roughness of the insert leads to a decrease of $\Delta {\bar{R}}_{a}$, i.e., the difference between the roughness of the molded part and the insert roughness is reduced. The opposite behavior can be observed when different thermoplastic materials are used (Figure 5b). For both types of PA6, the impression quality leads to negative but almost identical $\Delta {\bar{R}}_{a}$–values. However, for PA a disproportionate rise in the $\Delta {\bar{R}}_{a}$–value is the outcome of the MANOVA. Thus, the results of the conducted MANOVA confirm the assumption that the filler geometry and size have by far the strongest impact on impression quality.

**Figure 6:**
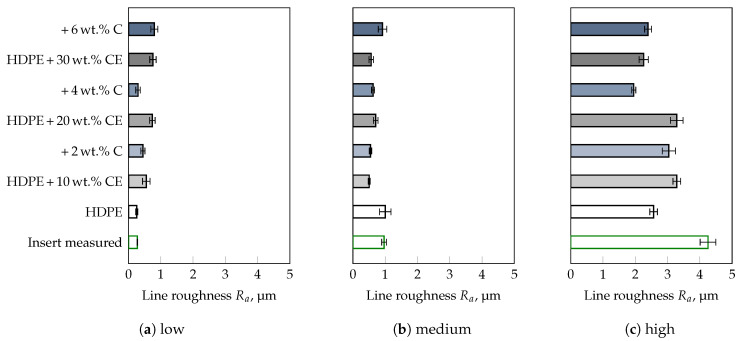
Influence of filler CalPlex Extra (CE, D50 = 0.7–0.90 μm), and compatibilizer (C) content on the impression quality. Deployed insert with a target line roughness of (**a**) 0.2 μm (low), (**b**) 0.8 μm (medium), and (**c**) 3.2 μm (high).

**Figure 7:**
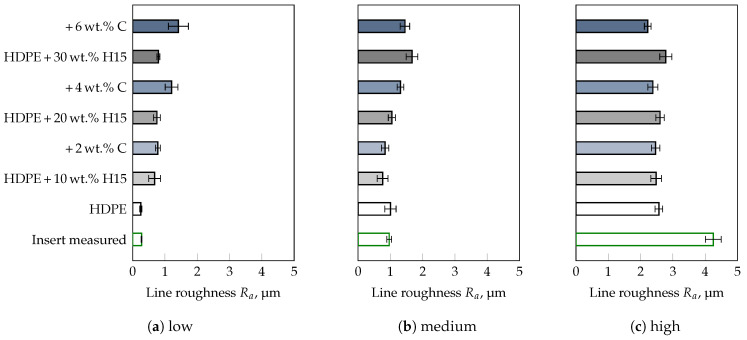
Influence of filler Plustalc H15 (H15, D50 = 5.4 μm), and compatibilizer (C) content on the impression quality. Deployed insert with a target line roughness of (**a**) 0.2 μm (low), (**b**) 0.8 μm (medium), and (**c**) 3.2 μm (high).

**Figure 8:**
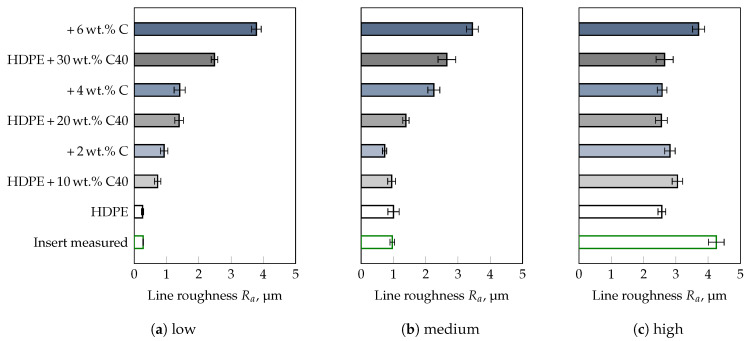
Influence of filler CalPlex 40 (C40, D50 = 16–25 μm), and compatibilizer (C) content on the impression quality. Deployed insert with a target line roughness of (**a**) 0.2 μm (low), (**b**) 0.8 μm (medium), and (**c**) 3.2 μm (high).

**Figure 9:**
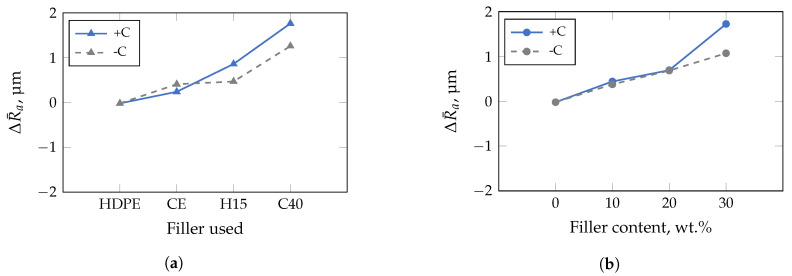
Results of multivariate analysis of variance performed with a confidence interval of 95% for insert with low roughness. Compatibilizer dependence is marked with +C (with), and −C (without). (**a**) An increasing filler diameter (D50-value) of filler CalPlex Extra (CE), Plustalc H15 (H15), and CalPlex 40 (C40) decreases the impression quality (higher $\Delta {\bar{R}}_{a}$-value). (**b**) Filler and compatibilizer content influences the impression quality (higher $\Delta {\bar{R}}_{a}$-value).

**Figure 10:**
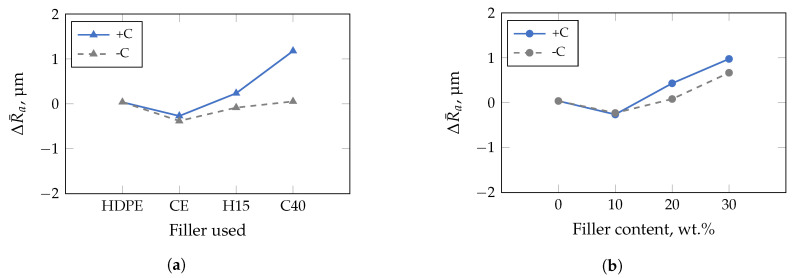
Results of multivariate analysis of variance performed with a confidence interval of 95% for insert with medium roughness. Compatibilizer dependence is marked with +C (with) and −C (without). (**a**) Combatibilizer increases $\Delta {\bar{R}}_{a}$ stronger in comparison to the filler used. CalPlex Extra (CE), Plustalc H15 (H15), and CalPlex 40 (C40). (**b**) Linear dependence of filler and compatibilizer content on impression quality.

**Figure 11:**
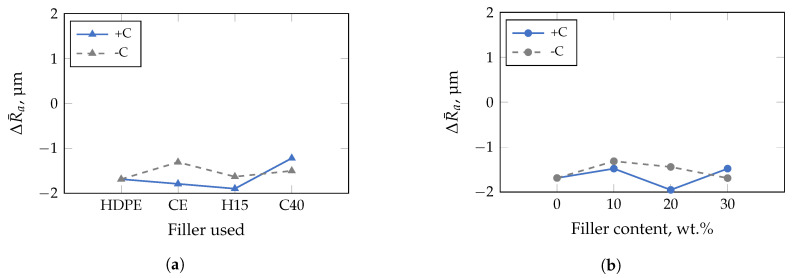
Results of multivariate analysis of variance performed with a confidence interval of 95% for insert with high roughness. Compatibilizer dependence is marked with +C (with) and −C (without). Although the used filler as well as the filler and compatibilizer content are statistically significant, no clear trend is discernible between (**a**) the filler CalPlex Extra (CE), Plustalc H15 (H15), CalPlex 40 (C40), and (**b**) the filler content.

To corroborate this statement, compounds were prepared from HDPE and three different fillers with known mean particle sizes, i.e., D50-value (cf. Table 1), followed by a comparison of results obtained for the commercial thermoplastics tested. Besides, the influence of a compatibilizer necessary for the distribution of the fillers in the polymer matrix was also taken into account. The results of the roughness measurements on the injection molded parts are shown for the filler CalPlex Extra in Figure 6, for Plustalc H15 in Figure 7, and for CalPlex 40 in Figure 8. As already shown in the preliminary investigations (see Figure 2b), the impression quality of the unfilled HDPE is excellent. This conclusion also includes inserts with low and medium roughness values, but does not apply to inserts with high $R_{a}$. Inserts with high $R_{a}$ cause impaired impression quality, i.e., the molded roughness of the part is lower than those of the insert, which will be discussed in more detail later. Furthermore, it is obvious that (i) the D50-value of the filler, (ii) the filler content, as well as (iii) the compatibilizer content affect the impression quality. Thus, more accurate conclusions cannot be provided, as these would be highly dependent on the person being assessed. To support the observations with statistical quantities, a MANOVA with a 95%-confidence interval was performed separately for each roughness class (low, medium, and high) of the insert as a first step. This enables the influences of filler type, weight percent of filler as well as of compatibilizer per roughness class to be considered independently of each other. After that, the three insert roughnesses will be included in the MANOVA to discuss a more general case. Within a 95%–confidence interval, the results illustrated in Figure 9, Figure 10 and Figure 11 are all statistically significant.

For the insert with low roughness (Figure 9), the impact of the median particle size in ascending order, i.e., corresponding to the filler type used, is shown in Figure 9a. With increasing filler size, e.g., D50-value of 0.75 μm–0.9 μm for CalPlex Extra (CE), 5.4 μm for Plustalc H15 (H15), and 16 μm–25 μm for CalPlex 40 (C40), $\Delta {\bar{R}}_{a}$ increases. The roughness of the component is only affected by the compatibilizer at D50-values of ≥5.4 μm (filler Plustalc H15). For lower D50-values of the filler, almost no difference between the specimens with and without compatibilizer is detectable. An increasing filler content causes a linear increase of $\Delta {\bar{R}}_{a}$, as shown in Figure 9b. Adding compatibilizer only affects the impression quality at a filler content of 30 wt.%. However, relative to the insert with low roughness, the results of the insert with medium roughness show a slightly different behavior (Figure 10). Even the addition of the filler with the lowest D50-value (CalPlex Extra) reduces the molded roughness, i.e., lower $\Delta {\bar{R}}_{a}$-values compared to the unfilled HDPE. As the D50-value increases, the $\Delta {\bar{R}}_{a}$ increases again linearly, but can not reach the $\Delta {\bar{R}}_{a}$-value of the unfilled HDPE. A similar behavior is achieved by adding the compatibilizer. Nevertheless, the increase in the molded roughness value for the samples with compatibilizer is more pronounced (Figure 10a). The same behavior can be observed during an increase of the filler content, whereby the change of $\Delta {\bar{R}}_{a}$ for the samples without compatibilizer is more prominent compared to the filler diameter (Figure 10b).

**Figure 12:**
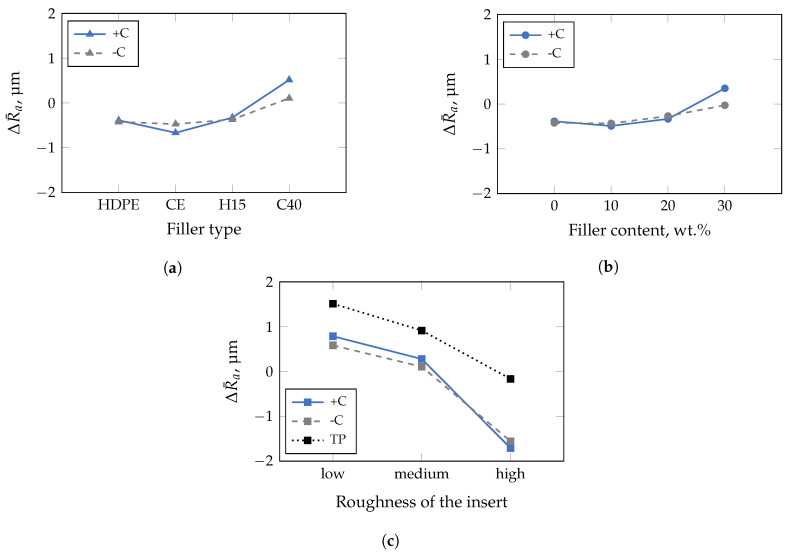
Results of multivariate analysis of variance performed with a confidence interval of 95% including different insert rougnesses. Compatibilizer dependence is marked with +C (with), and −C (without). (**a**) Combatibilizer impacts $\Delta {\bar{R}}_{a}$ stronger in comparison to the filler used. (**b**) The impression quality is influenced by the compatibilizer above a critical filler content. (**c**) A similar dependence, as observed in the studies of the commercial thermoplastic (TP) materials (cf. Figure 5a), is given for the filled samples with and without compatibilizer.

Both, the filler used as well as the filler and compatibilizer content affect the impression quality within a 95%-confidence interval of the conducted MANOVA significantly when applying the insert with the highest roughness (Figure 11). In addition, no clear trend could be confirmed, as the $\Delta {\bar{R}}_{a}$-values are within −1 μm and −2 μm. Accordingly, other influences on the impression quality must exist, which have not yet been taken into account. The results of the general case, i.e., when all studied insert roughnesses are considered in the MANOVA, are illustrated in Figure 12. In this case, even the unfilled HDPE is molded worse, i.e., recognizable by a negative $\Delta {\bar{R}}_{a}$-value (Figure 12a). In addition, a critical D50-value seems to exist, which impacts the impression quality, since $\Delta {\bar{R}}_{a}$ increases significantly with the addition of CalPlex 40 (highest D50-value). An analogy could also be observed in the preliminary investigations with PA, where the impression quality was almost not affected by the insert roughness applied (cf. Figure 4). Furthermore, the compatibilizer influence on the impression quality is more pronounced for fillers with high D50-values than for low ones. Similar relationships, as observed for the filler used, are valid for an increasing filler content (Figure 12b). The impact of the applied insert roughness on impression quality is shown in Figure 12c for the general case. As already discussed in Figure 5 for the commercial thermoplastic materials (black dotted line in Figure 12c), the trend of the impression quality is quite similar for the filled HDPE (gray dashed line). However, a significant shift in $\Delta {\bar{R}}_{a}$ is evident, resulting in smoother surfaces when using the fillers compared to the commercial thermoplastic materials, which are filled with unknown filler types. One reason for the shift in $\Delta {\bar{R}}_{a}$ could be the higher content of fillers (>47 wt.%), determined by TGA, and shown in Table 6. Another reason could be related to the fact that the commercial thermoplastics are filled with fillers having a significantly higher D50-value (>25 μm). Both factors have a strong influence on the $\Delta {\bar{R}}_{a}$-value, since the influence of the filler diameter and the filler content amplify each other. Furthermore, the addition of a compatibilizer increases the $\Delta {\bar{R}}_{a}$-value for the inserts with low and medium roughness but it causes the $\Delta {\bar{R}}_{a}$ for the insert with the highest roughness.

In general, the compatibilizer is chemically bonded to the filler, resulting in an expansion of the filler diameter, and D50-value, respectively [23]. Another indication that the commercial thermoplastics are composed of fillers with a higher D50-value, is that the addition of the compatibilizer causes a shift to higher $\Delta {\bar{R}}_{a}$-values, at least for inserts with low and medium roughness. So far, the only unanswered question is why the impression quality of the insert with the highest roughness follows a different trend compared to the inserts with low and medium roughness. Therefore, profile parameters like the average peak-to-valley height $R_{z}$, as well as the mean distances between the local (positive) peaks *S* of the measured line profiles of the several inserts will be discussed in more detail. As shown in Table 7, the inserts with low and medium roughness exhibit almost the same S-values, i.e., mean distance of local profile peaks *S*. However, the mean peak-to-valley height $R_{z}$ increases by a factor of about two. $R_{z}$-values in this range enable the unfilled HDPE to completely fill the valleys represented by *S* and $R_{z}$ before the flow front starts to solidify on the cavity wall, which is cold compared to the melt temperature.

Relative to the insert with the lowest roughness, the *S*-value of the insert with the highest roughness is 62.91 μm ± 3.03 μm, i.e., a factor of about four higher. In addition, this insert exhibits the highest $R_{z}$-value of 34.42 μm ± 1.78 μm, which corresponds to a factor of six compared to the insert with the lowest roughness. Because of this six times deeper valley, the HDPE tested will freeze at a selected mold temperature of 45 °C even before reaching the bottom of the valley, preventing a complete impression (cf. Figure 8c). An addition of fillers or compatibilizer to the HDPE generally does not significantly affect the molded roughness of the part (cf. Figure 11), because the applied fillers can penetrate the valley formed by *S* and $R_{z}$, since S≫D50 and S≫D98. In this case, the impression quality is primarily dominated by solidification processes of the polymer matrix.

For the inserts with low and medium roughness, the constraints S≫D50 and S≫D98 are only fulfilled for the filler CalPlex Extra. All other fillers showed significantly higher *D*-values (cf. Table 1), preventing the filler particles from entering the valleys. In addition, an increase in the filler content rises the material viscosity. This impairs the flow properties leading to an earlier solidification of the flow front. As a result, the filler geometry is more likely to be molded, leading to a complete independence between insert roughness and impression quality, as observed for instance, for PA (Figure 4) and HDPE + 30 wt.% C40 + 6 wt.% C (Figure 8) samples.

In order to suppress the premature freezing of the flow front, investigations would have to be carried out with higher mold and melt temperatures. However, since higher mold temperatures require longer process times, only the material viscosity of the individual formulations will be discussed in more detail. The objective is to demonstrate whether material formulations with identical viscosities yield the same impression quality for given and equal process conditions, i.e., based on the process simulation carried out. Assuming this, the viscosity curve of an unknown formulation might be shifted by means of temperature, i.e., WLF or Arrhenius temperature shift factor [42], to a known and well-working viscosity curve. In other words: if a perfect impression quality can be realized with material 1 but not with material 2, i.e., due to different material viscosities at given process conditions, the process temperature of the melt could be adjusted so that the viscosity of material 2 corresponds to that of material 1. This procedure would significantly reduce the effort associated with trial and error.

**Figure 13:**
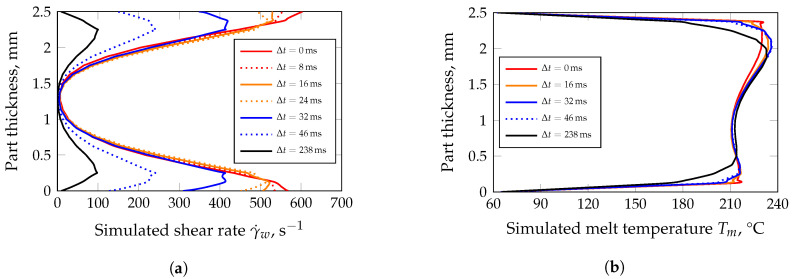
Simulated shear rates and temperature distributions 297 ms after the start of the filling phase (HDPE, center of the molded part). The time steps represent the decrease due to solidification processes at the cavity wall. Simulation parameters: (i) 20 elements in thickness direction, (ii) thermal conductivity of the mold $\lambda_{S}$ = 29 W m${}^{-1}$ K${}^{-1}$, and (iii) heat transfer coefficient between steel, and melt of α = 5 kW m${}^{-2}$ K${}^{-1}$. (**a**) Shear rates at the cavity wall decreases significantly after Δt = 24 ms. (**b**) Shear dissipation caused by the film gate leads to an increase in the average melt temperature of about 25 K.

**Figure 14:**
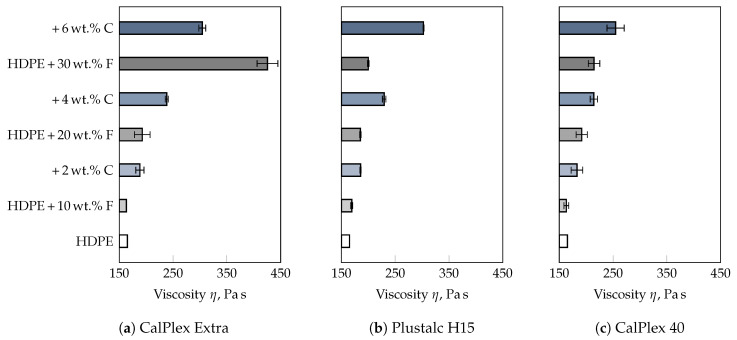
Viscosity values in the linear viscoelastic range (strain of 10%), obtained at a test temperature of 215 °C and a shear rate of 500 s${}^{-1}$. Fillers with (**a**) small diameters (CalPlex Extra) cause a more pronounced increase in viscosity compared to fillers with (**b**) medium (Plustalc H15) and (**c**) high (CalPlex 40) D50-values. Compatibilizer is marked as C and the filler as F.

For this purpose, the filling process was simulated with the unfilled HDPE by means of the simulation routine Moldflow^®^ Insight. The required material parameters of the HDPE are given in Appendix A. Thus, an estimation of the shear rate at the cavity wall ${\dot{\gamma }}_{w}$ and the corresponding melt temperature $T_{m}$ is possible. Both, ${\dot{\gamma }}_{w}$ and $T_{m}$ are subsequently the basis for the viscosity measurements. During the simulated filling process, the centre of the part on the film gate side was evaluated, as the roughness measurements were also carried out at this position (cf. Figure 1). After only 24 ms, ${\dot{\gamma }}_{w}$ drops significantly due to solidification processes (Figure 13a). In this context, it should be noted that surface structures in the order of magnitude of a few micrometres cannot yet be taken into account in simulation routines. Therefore, a shear rate of ${\dot{\gamma }}_{w}$ = 500 s${}^{-1}$ was chosen for the comparison of the viscosities of the different formulations. Figure 13b illustrates the distribution of the melt temperature as a function of time. Due to the narrow film gate of 0.5 mm, the material is extensively dissipated during the filling of the cavity, resulting in an average temperature increase of 25 K as well as a non-symmetrical distribution of the flow front temperature. Concerning the comparative viscosity measurements, the distribution of the mass temperature at the time step Δt = 0 s was averaged, resulting in a mean melt temperature of ${\bar{T}}_{m}$ = 215 °C. Figure 14 shows the viscosities determined at ${\bar{T}}_{m}$ and ${\dot{\gamma }}_{w}$ for all formulations. In general, the viscosity increases with the addition of filler or combatibilizer. However, the increase in viscosity is more pronounced for the filler with the lowest D50-value (CalPlex Extra, Figure 14a) compared to fillers with higher D50-values (Plustalc H15, Figure 14b, and CalPlex 40, Figure 14c). Moreover, it can be observed that the formulation with 10 wt.% filler, 10 wt.% filler and 2 wt.% compatibilizer as well as the formulation containing 20 wt.% Plustalc H15 as filler exhibit more or less the same viscosity (green viscosity values in Table 9). Consequently, as previously assumed, same viscosities should yield to an equal impression quality $\Delta {\bar{R}}_{a}$. At the same viscosity, the molded part is noticeably rougher with increasing filler diameter, even when an insert with low roughness is applied. The behavior is similar for the formulation with 30 wt.% filler and 6 wt.% compatibilizer when fillers like CalPlex Extra and Plustalc H15 are added to the polymer matrix (red viscosity values in Table 9).

On the basis of the investigations that were carried out, the assumption that an equal viscosity leads to an identical impression quality cannot be confirmed. In further studies, the focus should be on the mold temperature. An increased mold temperature shifts the contact temperature between the polymer melt and the mold surface towards the no-flow temperature, preventing a premature freezing of the polymer melt. This approach might be a possibility to significantly improve the impression quality.

## 4. Conclusions

The focus of this study was on investigating the influences, e.g., filler content, particle diameter of the filler, compatibilizer content as well as the roughness of the employed injection mold, on the impression quality $\Delta {\bar{R}}_{a}$. In this context, $\Delta {\bar{R}}_{a}$ is defined as the difference between the roughness of the molded part and the roughness of the mold insert. In order to identify possible influences on the impression quality, preliminary investigations were carried out with five commercially available thermoplastics and a mold without a defined roughness. Based on a mold surface roughness of $S_{a}$ = 0.26 μm, the unfilled HDPE could be molded very well. Compared to the HDPE ($S_{a}$ = 0.20 μm), the use of a highly filled polyamide (52 wt.%) resulted in a 27–times higher $S_{a}$-value. Additionally, it could be shown that almost the same filler content but different fillers, e.g., mineral and glass fibers, lead to a factor of two in impression quality. It can be assumed that the filler size and quantity are decisive for molding the cavity surface topography. Since the filler properties of commercially available thermoplastics are not specified by the manufacturer, different compounds with a known filler size were subsequently produced in-house. Finally, specimens were injection molded by employing spark eroded mold inserts with a target line roughness $R_{a}$ of 0.2 μm, 0.8 μm, and 3.2 μm.

To verify the suitability of the confocal microscope as well as to clarify the significant differences between target and measured roughness values of the employed inserts, measurements were carried out applying a VDI3400 reference gauge with defined $R_{a}$-values. As a result, the applied confocal microscope was suitable for the determination of the roughness. Furthermore, the difference between the target and measured insert roughness of about 30% did not originate from the applied confocal microscope. The difference could be explained by the fact that it is common practice in industry, after producing the desired insert roughness, e.g., by means of spark erosion, to check the roughness only with a VDI3400 reference gauge, i.e., yielding an accuracy within two VDI classes, or with portable hand-held devices. In general, it could be observed in the preliminary investigations that both, $S_{a}$ and $R_{a}$ agree very well if the specimens do not exhibit any curvature. In the case of curvatures due to shrinkage and warpage, it is mandatory to proceed with defined cut-off wavelengths and measuring lengths. To support the observations with statistical quantities, a multivariate analysis of variance was performed. As a result, a distinction must be made between two cases, whether it is possible for the unfilled matrix polymer to flow to the bottom of the valleys or not. If the flow front of filled thermoplastics does not solidify before reaching the bottom of the valleys, an increase of the filler diameter and of the filler content causes an increase of $\Delta {\bar{R}}_{a}$, i.e., the molded part becomes rougher compared to the selected insert roughness. Another influencing factor is related to the size of the filler, i.e., whether it fits into the valleys or not. However, if this is not the case, e.g., when using inserts with $R_{a}$-values lower than 0.8 μm and the filler CalPlex 40 with a D50-value of 16–25 μm, the geometry of the filler is primarily molded. Once the flow front solidifies before reaching the valleys, e.g., as observed for insert roughness of 3.2 μm, this generally leads to significantly smoother specimens. Any addition of filler as well as a change in the quantity of filler or compatibilizer reveals no significant effect on the impression quality. In order to improve the impression quality in this case, the occurring melt temperature and the wall shear rate during the filling process was calculated on the basis of a filling simulation. Subsequently, viscosity measurements were carried out applying the simulated parameters with the objective of correlating the impression quality with the material viscosity. On the basis of these investigations, the assumption that an equal viscosity at given process conditions leads to an identical impression quality could not be confirmed.

In ongoing studies, the focus should be on the mold temperature. An increased mold temperature shifts the contact temperature between the polymer melt and the mold surface towards the no-flow temperature, preventing a premature freezing of the polymer melt. This approach might be a possibility to significantly improve the impression quality, when filled polymers will be applied.

## Figures and Tables

**Table 1:** Physical properties of the applied fillers.

Filler	Top Cut	Median Particle Size
#	*D*98, μm	*D*50, μm
CalPlex Extra (CE)	3.50	0.75–0.90
Plustalc H15 (H15)	18.0	5.4
CalPlex 40 (C40)	90–150	16–25

**Table 2:** Process settings for the in-house production of different formulations on the twin screw extruder.

Temperature of the barrel, °C	155 *	160	165	170	175	180	180	185	185	190	180 **
Screw rotational speed, min${}^{-1}$	400										

* Hopper, ** Die.

**Table 3:** Formulation of the in-house produced compounds.

Filler	Compatibilizer
wt.%	wt.%
10	0
	2
20	0
	4
30	0
	6

**Table 4:** Process settings on the injection molding machine.

	HDPE-Matrix	PA6 (MF + GF)	PA6 (MF)	PC	PA
Barrel temperature, * °C	175–190	240–260	240–260	300–320	240–260
Screw rotational speed, min${}^{-1}$	50	75	75	75	75
Back pressure, MPa	10	2.5	2.5	10	2.5
Screw position after dosing, mm	28	38	38	38	38
Injection rate, mm s${}^{-1}$	50 ^i^	75 ^ii^	120 ^iii^	75 ^iv^	75 ^ii^
Switchover point, mm	9	4.7	5.5	5	4.7
Holding time, s	2/8/2	10	10	10	10
Holding pressure, MPa	25/50/35	130	110	80	70
Temperature of the mold, °C	45	80	80	80	80
Residual cooling time, s	10	15	15	10	15

* From hopper to die in 5 K steps. (i) For the last 4 mm the injection rate was set to 20 mm s${}^{-1}$. (ii) For the last 2.3 mm the injection rate was set to 10 mm s${}^{-1}$. (iii) For the last 3.5 mm the injection rate was set to 10 mm s${}^{-1}$. (iv) For the last 2 mm the injection rate was set to 10 mm s${}^{-1}$.

**Table 5:** Recommended cut-off wavelength $\lambda_{c}$ for non-periodic profiles depending on the expected line roughness $R_{a}$ according to ISO 4288 [37].

Line Roughness $R_{a}$	Cut-Off Wavelength $\lambda_{c}$
μm	mm
0.1–2	0.8
2–10	2.5

**Table 6:** Filler content of commercial thermoplastic materials determined by TGA.

Material	Decomposition	Content of Filler
#	wt.%	wt.%
HDPE	99.2	0
PA6 (MF+GF)	50.2	49.8
PA6 (MF)	52.6	47.4
PC	63.9	36.1
PA	47.9	52.1

**Table 7:** Surface parameters of the applied inserts.

Surface Parameter	Insert Roughness		
	Low	Medium	High
Target roughness $R_{a}$, μm	0.20	0.80	3.20
Line roughness $R_{a}$, μm	0.28 ± 0.01	0.97 ± 0.07	4.25 ± 0.24
Average peak-to-valley height $R_{z}$, μm	5.39 ± 0.02	10.10 ± 1.41	34.42 ± 1.78
Mean distance of local profile peaks *S*, μm	16.90 ± 0.32	15.89 ± 0.55	62.91 ± 3.03

**Table 8:** Proof of the suitability of the confocal microscope in terms of reproducibility.

VDI 3400 Reference Gauge	Target Line Roughness	Measured Line Roughness *	Standard Deviation		Deviation to VDI 3400
**#**	μm	μm	μm	%	%
00	0.10	0.09	0.01	10	−13
03	0.15	0.13	0.01	10	−16
06	0.20	0.22	0.02	7	+9
15	0.55	0.47	0.01	2	−15
18	0.80	0.78	0.06	8	−3
21	1.10	1.20	0.14	12	+9
24	1.60	1.72	0.13	8	+7
30	3.20	3.57	0.22	7	+12

* Mean value of five measuremets.

**Table 9:** Correlations of viscosity values η and impression quality $\Delta {\bar{R}}_{a}$ of the insert with low roughness for selected formulations. Viscosities were determined in the linear viscoelastic range (strain of 10%) at a test temperature of 215 °C and a shear rate of 500 s${}^{-1}$. Compatibilizer is marked as C and the filler as F.

Formulation	CalPlex Extra		Plustalc H15		CalPlex 40	
**HDPE**	**η, Pa s**	**$\Delta {\bar{R}}_{a}$, μm**	**η, Pa s**	**$\Delta {\bar{R}}_{a}$, μm**	**η, Pa s**	**$\Delta {\bar{R}}_{a}$, μm**
+10 wt.% F + 2 wt.% C	188 ± 8	0.17	186 ± 1	0.51	183 ± 11	0.65
+20 wt.% F	193 ± 15	0.47	186 ± 1	0.48	192 ± 11	1.11
+30 wt.% F + 6 wt.% C	304 ± 6	0.53	303 ± 1	1.14	255 ± 16	3.51

η: mean value of three measuremets.

**Figure A1:**
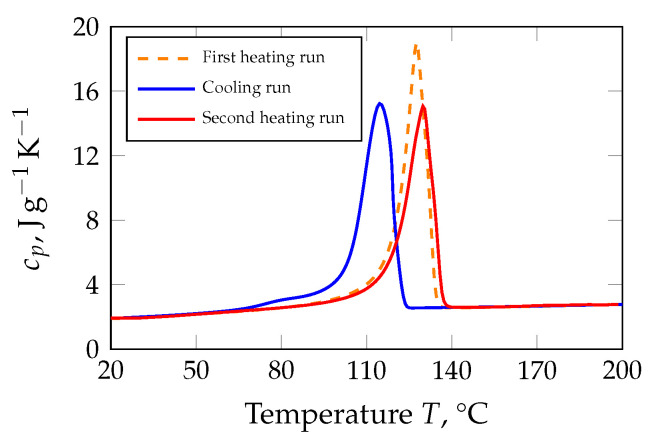
Calculated specific heat capacity $c_{p}$ of HDPE at a heating rate of 10 K min${}^{-1}$ and at a cooling rate of 20 K min${}^{-1}$.

## Data Availability

All data presented are only available after request from the corresponding author assuming a formal approval of our company partners.

## Abbreviations

The following abbreviations are utilized in this manuscript:

*A*	Mapped area by vectors x and y
$A_{i}$	Data-fitted coefficients of the Cross-WLF model
*B*	Pressure sensitivity of the material
$b_{i}$, $b_{i_{m}}$, $b_{i_{s}}$	Approximation parameters of the two-domain pvT-model of Tait
C	Compatibilizer
C40	CalPlex 40
CE	CalPlex Extra
c	Constant
$c_{p}$	Specific heat capacity
*D*	Diameter
$D_{i}$	Data-fitted coefficients of the Cross-WLF model
D50	Median particle size
D98	Top cut
DSC	Differential scanning calorimetry
F	Filler
FRT	Confocal microscope of Fries Research and Technology GmbH.
GF	Glass fiber
H15	Plustalc H15
HDPE	High density polyethylene
j	Number of peaks
L	Length
l	Measuring length
MANOVA	Multivariate analysis of variance
MF	Mineral filler
m	Melt
n	Power law index
PA	Polyamide
PC	Polycarbonate
PE-g-MA	Maleic anhydride functionalized high density polyethylene compatibilizer
p	Pressure
pvT	Pressure-specific volume-temperature
$R_{a}$	Line roughness
$R_{z}$, $R_{z_{i}}$	Peak-to-valley height of the profile
S	Mean distance between the local (positive) profile peaks
$S_{a}$	Surface roughness
s	Solid
T	Temperature
TGA	Thermogravimetric analysis
$T_{m}$	Melt temperature
$T^{*}$	Pressure-dependent transition temperature
${\bar{T}}_{m}$	Melt mean temperature
$T_{t}$	Transition temperature
*v*	Specific volume
$v_{0}$	Specific volume at zero pressure
$v_{t}$	Specific volume decrease due to crystallization
WLF	Williams-Landel-Ferry
wt.%	Weight percent
z(x), z(x,y)	Measured roughness profile
$\Delta {\bar{R}}_{a}$	Difference in line roughness between the part and the applied insert
Δt	Time step
α	Heat transfer coefficient
$\dot{\gamma }$	Shear rate
${\dot{\gamma }}_{w}$	Shear rate at the cavity wall
η	Melt viscosity
$\eta_{0}$	Zero shear viscosity
λ	Thermal conductivity of polymer
$\lambda_{c}$	Cut-off wavelength
$\lambda_{S}$	Thermal conductivity of mold steel
$\tau^{*}$	Critical shear stress

## Appendix A

All necessary material data for a filling simulation were determined for the HDPE according to the methods described in Section 2.5. The applied approximation models as well as material properties are shown below.

The viscosity parameters given in Table A1 were fitted with the Cross-Williams-Landel-Ferry (Cross-WLF) model [28,29]:

(A1) $$\eta =\frac{\eta_{0}}{1+{\left(\frac{\eta_{0}\dot{\gamma }}{\tau^{*}}\right)}^{1-n}}\;,$$

where η is the melt viscosity, $\eta_{0}$ represents the zero shear viscosity, $\dot{\gamma }$ is the shear rate, $\tau^{*}$ is the critical shear stress level, and *n* is the power law index. In addition, the zero shear viscosity $\eta_{0}$ can be modeled with the WLF equation:

(A2) $$\eta_{0}=D_{1}\exp\left[-\frac{A_{1}\left(T-T^{*}\right)}{A_{2}+\left(T-T^{*}\right)}\right]\;,$$

(A3) $$T^{*}=D_{2}+D_{3}p\;,$$

(A4) $$A_{2}=A_{3}+D_{3}p.$$

Here, *T* is the temperature, $T^{*}$ represents a transition temperature depending on pressure *p*, $D_{3}$ provides a description of the pressure dependence, and $D_{1}$, $D_{2}$, $A_{1}$, $A_{2}$, as well as $A_{3}$ are data-fitted coefficients.

**Table A1:** Cross-WLF approximation parameters of the applied viscosity model.

Parameter		
*n*	0.401	-
$\tau^{*}$	6273	Pa
$D_{1}$	$1.756\times 10^{-8}$	Pas
$D_{2}$	208.95	K
$D_{3}$	0 *	$K{\mathrm{Pa}}^{-1}$
$A_{1}$	15.740	-
$A_{2}$	377.77	K

* No data for pressure dependence available.

Figure A1 provides the specific heat capacity calculated from the DSC measurements.

Measured pvT-data (see Table A2) were approximated with the two-domain pvT-model of Tait [32,33]:

(A5) $$v=v_{0}\left[1-c\log\left(1+\frac{p}{B}\right)\right]+v_{t}\;,$$

where *v* is the specific volume at a pressure *p* and at a temperature *T*, $v_{0}$ is the specific volume at zero pressure and only dependent from T, *c* is a constant with the value 0.0894, *B* accounts for the pressure sensitivity of the material, and $v_{t}$ is the specific volume decrease due to crystallization. Both, $v_{0}$ and *B* need to be calculated for the solid (s) and melt (m) regions separately:

(A6) $$v_{0}=b_{1_{s,m}}+b_{2_{s,m}}\left(T-b_{5}\right)\;,$$

(A7) $$B=b_{3_{s,m}}\exp\left[b_{4_{s,m}}\left(T-b_{5}\right)\right]\;.$$

The distinction between the solid and melt region is made under consideration of the transition temperature $T_{t}$:

(A8) $$T_{t}=b_{5}+b_{6}p\;.$$

For semi-crystalline materials, $v_{t}$ can be expressed as:

(A9) $$v_{t}=b_{7}\exp\left[b_{8}\left(T-b_{5}\right)-b_{9}p\right]\;.$$

Here, $b_{1}$–$b_{9}$ are approximation parameters obtained from at least three measured pressure levels.

**Table A2:** Approximation parameters for HDPE in the solid (s), melt (m), and transition region of the two-domain pvT-model of Tait.

Solid			Melt		
$b_{1s}$	$1.046\times 10^{-3}$	$m^{3}K^{-1}$	$b_{1m}$	$1.250\times 10^{-3}$	$m^{3}{\mathrm{kg}}^{-1}$
$b_{2s}$	$1.090\times 10^{-8}$	$m^{3}{\mathrm{kg}}^{-1}K^{-1}$	$b_{2m}$	$8.634\times 10^{-7}$	$m^{3}{\mathrm{kg}}^{-1}K^{-1}$
$b_{3s}$	$4.477\times 10^{8}$	Pa	$b_{3m}$	$9.313\times 10^{7}$	Pa
$b_{4s}$	$1.403\times 10^{-4}$	$K^{-1}$	$b_{4m}$	$2.273\times 10^{-3}$	$K^{-1}$
$b_{7}$	$1.887\times 10^{-4}$	$m^{3}{\mathrm{kg}}^{-1}$	**Transition**		
$b_{8}$	$3.977\times 10^{-2}$	$K^{-1}$	$b_{5}$	$3.995\times 10^{2}$	K
$b_{9}$	$1.255\times 10^{-8}$	${\mathrm{Pa}}^{-1}$	$b_{6}$	$2.800\times 10^{-7}$	$K\;{\mathrm{Pa}}^{-1}$

Finally, the thermal conductivity is given in Table A3.

**Table A3:** Measured thermal conductivity of the HDPE.

Temperature	Thermal Conductivity
°C	W m${}^{-1}$ K${}^{-1}$
27.6	0.283
60.7	0.274
178.6	0.225
197.5	0.238
